# Recent Advances in Superhydrophobic Surfaces and Applications on Wood

**DOI:** 10.3390/polym15071682

**Published:** 2023-03-28

**Authors:** Xinyue Wei, Xiaoting Niu

**Affiliations:** College of Materials Science and Engineering, Northeast Forestry University, Harbin 150040, China

**Keywords:** superhydrophobic surfaces, wood, functionality, applications

## Abstract

Superhydrophobic substances were favored in wood protection. Superhydrophobic treatment of wood is of great significance for improving the service life of wood and expanding its application fields, such as improving dimensional stability, durability, UV stability, and reducing wetting. The superhydrophobic phenomenon is attributed to the interaction of micro/nano hierarchical structure and low surface energy substances of the wood surface. This is the common method for obtaining superhydrophobic wood. The article introduces the common preparation methods of superhydrophobic wood material coatings and their mechanisms. These techniques include lithography, sol–gel methods, graft copolymerization, chemical vapor deposition, etc. The latest research progress of superhydrophobic wood material coatings application at domestic and overseas is reviewed, and the current status of superhydrophobic coating application in wood materials and construction is summarized. Finally, superhydrophobic on wood in the field of applied research is presented, and the development trend in the field of functional improvement of wood is foreseen.

## 1. Introduction

As a typical natural material with the characteristic of carbon fixation during its life cycle, renewable and environmentally friendly, wood has been widely applied in areas of building, furniture, and energy generation as well as other functional materials due to its hierarchical and porous structure. However, due to the hydrophilic nature of wood, it has a tendency towards hygroexpansion, which affects its dimensional stability and durability and limits its practical application range. Therefore, it is necessary for wood to be modified against water to prolong its service life and broaden its application area.

In recent years, with further research on superhydrophobic coatings, the multifunctional coatings achieved via numerous methods have become a trend. Multifunctional coatings achieved by many methods have become more visible in various fields [1,2]. The application of superhydrophobic coatings in the field of wood materials has been widely studied. Wood superhydrophobic surfaces are constructed mainly by different methods of forming rough structures and functionalized with low surface energy substances, such as sol–gel, lithography, and so on. Functionalization of wood surfaces improves and enhances the properties of wood, including flame retardancy, dimensional stability, and abrasion resistance, and endows wood with new biomimetic functions, such as self-cleaning, superhydrophobicity, phase change energy storage, and so on [3,4,5,6]. Researchers have developed a sustainable method to fabricate a highly compressible wood sponge-based sensor with good water resistance and fast electrical response for human motion detection [7]. The wood sponge-based sensor exhibits superhydrophobicity with a WCA of 152°, maintaining its superhydrophobic state even after 100 compression cycles. In addition, the sensor had high sensitivity, fast response time, and strong fatigue resistance, capable of undergoing 1000 compression cycles at 60% strain with no change in resistance response. The sensor has been successfully applied to detect a variety of physical activities including joint flexion, walking, running, and squatting, but also subtle movements such as voice recognition and wrist pulses. The wood sensor also holds great promise for applications in wearable electronics, artificial intelligence, and electronic skin. It has a broad application prospect. It is necessary to build superhydrophobic surfaces for the effective development and utilization of wood resources and to further broaden the application of wood in environmental protection, intelligent construction, chemical, and other fields. Wood products with superhydrophobic properties will be greatly appreciated as high-value-added products by a more discerning and demanding consumer market.

Superhydrophobic wood coatings with environmental friendliness are becoming a mainstream direction for popular research [8]. Wax-based materials have been widely used as environmentally friendly materials for the preparation of superhydrophobic wood coatings [9]. Ideally, wood finishes should provide efficient protection from water while retaining the overall bio-based character of the material. In order to meet this challenge, superhydrophobic coatings on the wood are achieved by using materials that are environmentally friendly. It is in line with today’s social theme of “green and sustainable development” to use green, renewable, and biodegradable resources to promote human social development. 

This paper mainly reviews the latest research progress of domestic and foreign superhydrophobic wood materials coatings. Based on the basic principle of superhydrophobic coating constructed on the surface of wood, this paper summarizes the latest research status, the common methods, and application prospects of superhydrophobic coatings on wood, which will make an important contribution to the potential multifunctional application development of superhydrophobic coatings in the field of wood and intelligent buildings in the future.

## 2. The Preparation of Superhydrophobic Coatings on Wood

It was defined as superhydrophobic surfaces if the static water contact angle (WCA) was greater than 150° and dynamic sliding angle (SA) was less than 10° [10,11]. Superhydrophobic materials have many exceptional properties such as self-cleaning, oil–water separation capacity, resistance to icing, corrosion, as well as fouling, which enable various applications in surface engineering, food engineering, photothermal conversion, and microfluidic control, etc. [12,13,14,15]. Superhydrophobicity is common in the natural world and is found in lotus leaves, rose petals, peanut leaves, butterfly wings, water striders, fish scales, and gecko feet [16]. The method of preparing superhydrophobic coatings is usually also applicable to the construction of superhydrophobic surfaces on wood and other materials. Micro/nano-hierarchical roughness and low surface energy are critical for fabricating superhydrophobic surfaces. Based on this, the common methods used in the preparation of superhydrophobic wood materials include: lithography, sol–gel methods, graft copolymerization, chemical vapor deposition, hydrothermal, etching, etc.

### 2.1. Lithography

The lithography method refers to filling the substrate material with nano or micron structure by nanoimprint lithography or slip casting, thus obtaining a superhydrophobic surface. Template materials are commonly used to build micro and nanostructured superhydrophobic surfaces, including natural materials and artificial materials. Natural substrates with superhydrophobic structures such as lotus leaves and rose petals. The other is an artificial substrate that replicates the superhydrophobic structure by mechanical processing or etching, of which polyvinyl alcohol (PVA), and polydimethylsiloxane (PDMS) is the most widely used. The advantages of the lithography method include short manufacturing time and low cost, applicability to extensive materials, simplicity of operation, and availability for mass production.

**Figure 1:**
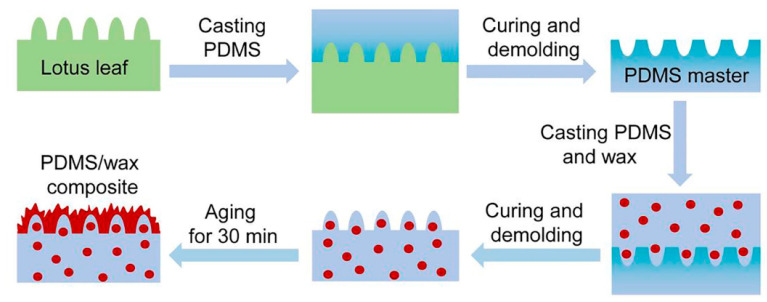
Schematic showing the preparation procedure of the self-healing superhydrophobic surface. Reproduced from [17], with permission from Elsevier, 2020.

The fabrication procedure of the self-healing superhydrophobic surfaces was shown in Figure 1 [17]. The superhydrophobic n-nonadecane material was prepared by secondary replication using fresh lotus leaf as the original template material and PDMS as the medium to transcribe the superhydrophobic surface. Finally, the rough structure of the lotus leaf surface was successfully replicated on the polymer surface. By aging the as-prepared sample at room temperature for more than 30 min, n-nonadecane wax would migrate from the matrix to the surface to form a steady equilibrium morphology, and the self-healing superhydrophobic surface was thus obtained. It was found that the contact angle (WCA) on the material surface reached 161.4°.

**Figure 2:**
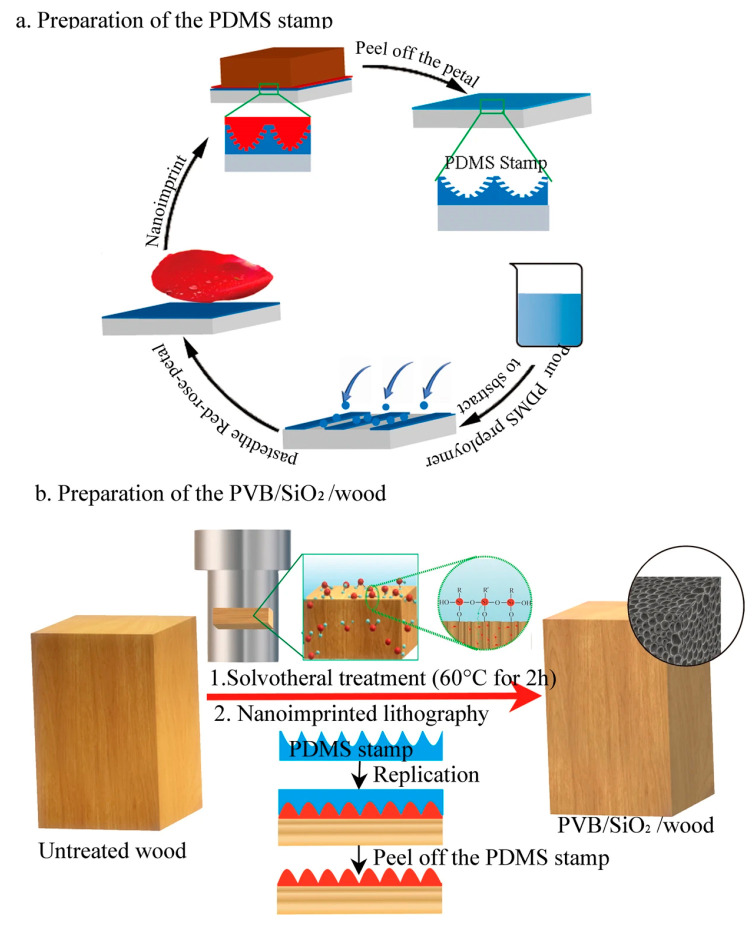
Schematic illustration of (**a**) the replication process of the PDMS stamp with negative nanostructures of biomimetic red rose petals, (**b**) the fabrication process for PVB/SiO_2_/wood [18].

Similarly, it was reported that a petal-like PVB/SiO_2_ superhydrophobic wood was successfully fabricated by using red rose petals, using rose petals as the base material, and cross-linked PDMS was used as the master template and stamp for nanoimprinted lithography in Figure 2 [18]. Then, the polyvinyl butyral (PVB) mixture solution was evenly coated on the wood surface, and the rough structure on the PDMS master was successfully printed onto the wood surface using the secondary replication method, and finally the superhydrophobic wood with the same structure as the rose petal was obtained. The as-prepared PVB/SiO_2_/wood surface not only had robust superhydrophobic performance during the ultrasonic rinse and sand abrasion tests, but also stable super-repellency towards commonly used liquids, including brine, tea, milk, and vinegar, and improved the dimensional stability, which can satisfy our daily life application needs. By the same token, PDMS stamps created the hierarchical structures similar with taro leaf surface when the stamps were replicated from fresh taro leaves by soft lithography [19]. Fe_3_O_4_ nanoparticles were mixed into PDMS suspensions to obtain Fe_3_O_4_/PDMS suspensions, after being dried and PDMS stamps were peeled off, biomimetic wood surface with superhydrophobic and superparamagnetic was created. It is a feasible method for constructing naturally biomorphic structures on wood surfaces with tailored functions. The researchers studied that a new superhydrophobic bamboo timber could promote the applications of superhydrophobic and conductive bamboo products [20]. Superhydrophobic bamboo timber was fabricated by magnetron sputtering and nanoimprinting techniques. Conductive copper films were deposited on the bamboo surface, and then the lotus leaf structure pattern was transferred to the copper bamboo surface. The prepared surface with the lotus leaf structure showed superhydrophobic properties with a water contact angle of 152° and a sliding angle of only 5°. The mechanical stability of the prepared superhydrophobic bamboo was assessed qualitatively by sand abrasion tests. An amount of 20 g of sand grains with diameters ranging from 100 to 300 μm was impacted onto the surface from a height of 30 cm. The surface retained its superhydrophobicity after 100 tests. The coating exhibited good mechanical resistance, environmental stability, and high electrical conductivity. 

### 2.2. Sol–Gel Methods

The sol–gel method is a reaction process that generates nanoscale rough structures on the wood surface and is a common method to obtain superhydrophobic surfaces. Usually, impregnation of wood in treated superhydrophobic composite solvent adsorbs nanoparticles from the solution on the wood surface, normally containing chemically stable oxides such as silicon, zinc, and aluminum [21], then obtains a stable transparent sol through a series of chemical reactions such as hydrolysis and condensation. The solute forms a gel with a three-dimensional spatial network structure by solvent evaporation or by heating and drying. Thus, generating a nano-scale rough structure on the wood surface by the reaction process, is a common method to obtain superhydrophobic surfaces. Preparation of stable, durable, and cost-effective superhydrophobic coatings by combining sol–gel method with other methods in order to improve the durability of coatings [22,23,24,25,26].

**Figure 3:**
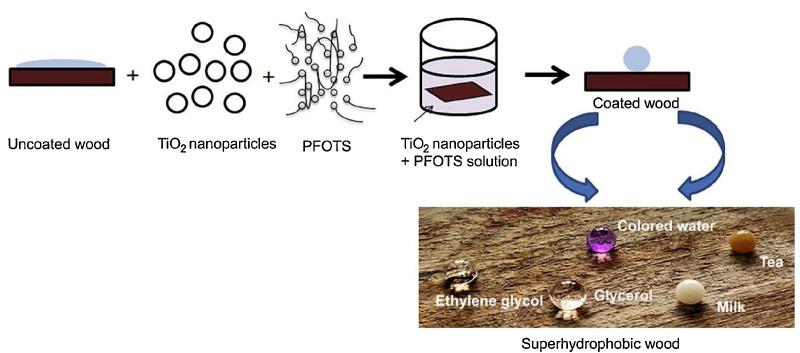
Schematic representation of synthesis of superhydrophobic wood surface. Reproduced from [27], with permission from Elsevier, 2020.

The TiO_2_ nanoparticles were added to the perfluorooctyltriethoxysilane (PFOTS) solution, hydroxyl group present on TiO_2_ nanoparticle reacts with the hydrophilic head (-Si(OC_2_H_5_)_3_) of PFOTS molecule which functionalizes the TiO_2_ nanoparticles [27], formed a uniform self-assembled coating on the wood surface, and the product adheres to the wood surface, leading to the hydrophobic end (C_8_H_4_F_13_) upward, giving the wood surface superhydrophobic properties in Figure 3. During the sol–gel process, nanoparticles are deposited on the wood surface, which often leads to poor coating stability and weak interfacial bonding ability. In order to improve the bonding strength between the sol–gel solution and the substrate material, enhance the interfacial bonding, and prepare materials with mechanical robustness and non-wetting properties. So, the researchers employed Ti–Si sol to build the rough microstructure and polydimethylsiloxane (PDMS) as low surface energy to cooperative fabricate the superhydrophobic wood [28]. Because of the formation of Si–O–Ti, Si–O–Si, and Ti–O bonds through -OH and polycondensation between hydroxyl groups, the cross-linked network of SiO_2_ and TiO_2_ was constructed. Further, the PDMS polymer chains were covalently attached to the Si–Ti cross-linked network by graft polymerization of KH-132, forming a low surface energy structure to fabricate a Ti–Si@PDMS superhydrophobic modification system. Enhanced interfacial bonding is due to the presence of covalent bonds. After surface carbonization treatment, the successful transfer of Ti–Si@PDMS from inside the wood by the thermally induced response achieved superhydrophobic self-healing.

In addition, it is essential to find efficient and stable sol–gel preparation methods. The researchers combined the sol–gel method with electron beam (EB) curing technology [29]. Robust superhydrophobic layers with hierarchical micro/nano-roughness structures were modified on the wood surface by in situ mineralization and polymerization. PDMS and cross-linked monomers (MAPS) formed new covalent bonds between TiO_2_ and the wood substrate after EB radiation. The wood exhibited strong superhydrophobicity and excellent UV resistance. The prepared wood exhibited a water contact angle of about 165.7° and was significantly repellent to many aqueous phase liquids, such as cola, strongly acidic, alkaline droplets, etc. Furthermore, excellent mechanical durability and UV resistance are provided. After 18 days of accelerated UV aging tests, its exceptional repellency to water was maintained. Superhydrophobic wood shows its potential application in furniture and construction.

The reaction process is easy to control and operate, and the homogeneity of the prepared samples is excellent for the preparation of superhydrophobic materials using sol–gel method. Enhancing the bonding strength between superhydrophobic surface and substrate and improving the functionalization of sol–gel method by compounding solvents so that the prepared materials can achieve both superhydrophobic properties and good thermal stability and mechanical properties. Yet, the price of raw materials is relatively expensive, usually, the whole sol–gel process takes a long time, and often needs a few days or a few weeks. If improvement is made on this basis it can shorten the reaction time. Sol–gel method will be the key research direction in the future.

### 2.3. Graft Copolymerization

Graft copolymerization is the grafting of low surface energy substances on the material surface in the form of covalent bonds through chemical reactions, thus improving the hydrophobicity of the material [30,31]. The surface of wood contains a large number of reactive groups such as hydroxyl and carboxyl groups, which create the necessary conditions for graft copolymerization reactions. The presence of covalent bonds makes the superhydrophobic coating stable in the graft copolymerization process, but this method takes longer preparation time and production is costly.

**Figure 4:**
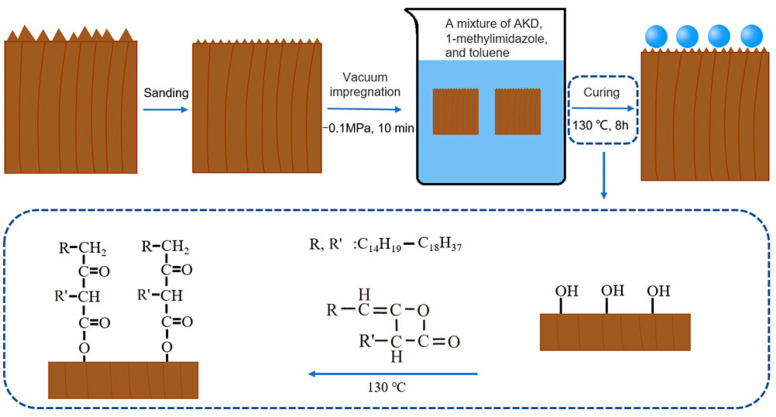
Schematic of the graft copolymerization reaction between the AKD and wood [32].

To improve the hydrophobicity of hardwoods, according to the research of Cao et al. [32], the sapwood of birch, oak, and black walnut, was modified with grafted alkylenone dimer (AKD), as shown in Figure 4. After AKD modification, the hydroxyl group reacts with AKD to form a hydrophobic layer. The reaction forms β-ketoester bonds that reduce the free energy of the wood surface. AKD-modified wood exhibits a high degree of hydrophobicity, as well as excellent resistance to water, acids, and toluene, and self-cleaning effects. Yang et al. [33] constructed micro/nanostructures using PDA and alumina nanoparticles (Al_2_O_3_). The superhydrophobic wood was prepared through grafting low surface energy to the wood surface modified by octadecyltrimethoxychlorosilane The superhydrophobic wood surfaces exhibited excellent resistance to flow scouring, acid and alkali corrosion, and organic solvents, providing excellent stability in the face of environmental challenges. Based on conventional grafting methods, atomic transfer radical polymerization (ARGET ATRP) is a low-cost reactive polymerization with surface initiating activator (ARGET ATRP) regenerated by electron transfer to efficiently generate homogeneous and dense polymers [34]. This approach improves the stability of superhydrophobic surfaces and has good industrial prospects. In a study [35], ash wood was modified by regenerating surface initiating activators through electron transfer atom radical polymerization at low catalyst concentrations using elemental silver as a medium. The hydrophobic and antimicrobial properties of the wood were obtained by functionalizing ash wood with poly(methyl methacrylate) (PMMA) and poly(2-(dimethylamino)ethyl methacrylate) (PDMAEMA). This study improves the wood properties from an eco-economic point of view and offers great opportunities for its use in industry and in furniture production.

### 2.4. Chemical Vapor Deposition

The CVD method [36] is a modification method to achieve a modification effect by reacting certain substances in the solution with the modified material to form a new substance through a gas-phase reaction to build a roughness or a nano-scale thin film material on the surface of the wood material. The CVD method is a low-cost, simple, and effective method for the preparation of hierarchical micro- and nano-rough structures. However, the demanding harsh reaction conditions of the CVD method have limited the large-scale application in the preparation of superhydrophobic wood.

**Figure 5:**
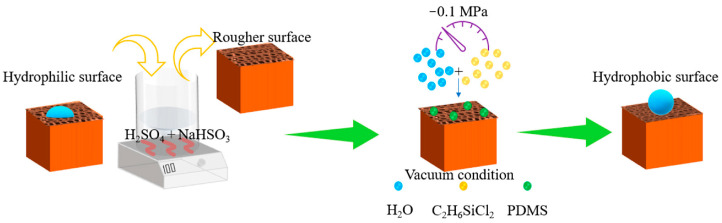
Schematic illustration of description of the entire process [38].

They were used to prepare stable superhydrophobic coatings on wood surfaces that mussel-inspired polydopamine (PDA) chemistry and electroless deposition approaches. The as-formed PDA coating on a wood surface exhibited a hierarchical micro/nano roughness structure and functioned as an “adhesive layer” between the substrate and a metallic film by the metal chelating ability of the catechol moieties on PDA, allowing for the formation of a well-developed micro/nanostructure hierarchical roughness [37]. Additionally, the coating acted as a stable bridge between the substrate and hydrophobic groups. The prepared superhydrophobic materials exhibit excellent stability under UV aging, ultrasonic cleaning, impregnation with strong acids, bases, and organic solvents, and boiling of water at high temperatures. In comparison, Yang et al. [38] prepared hydrophobic polydimethylsiloxane-coated wood with a water contact angle of 157.3° by applying low-temperature chemical vapor deposition technique in Figure 5. During the study, in order to quantify the mechanical stability of the hydrophobic wood surface, a sandpaper (1500 mesh) friction test was used to assess the wear resistance of the hydrophobic coating on the wood surface. Prior to the sandpaper friction test, there was a lot of particulate matter adhering to the wood cavity, while the hydrophobic coating was still observable after the wear test. The microstructure of the hydrophobic surface of the wood was slightly damaged. This clearly showed that the surface of PDMS@wood remains hydrophobic even after severe abrasion. Dichlorodimethylsilane was used as a chemical material with enhanced thermal stability of wood and superhydrophobicity was maintained in the long term. Similarly, Ma et al. [39] constructed a nanocellulose-derived superhydrophobic self-cleaning coating with CaCO_3_ particles by chemical vapor deposition (CVD), dispersed CaCO_3_ particles and PDMS into THF through spraying. Then, chemical vapor deposition occurred at 105 °C. The contact angle is more than 150° of the treated superhydrophobic surface and with a minimum sliding angle of 6.4°. Low-cost CaCO_3_ nanoparticles were found to confer a self-cleaning function.

### 2.5. Hydrothermal Method

The hydrothermal method [40,41] involves the reaction of insoluble substances to produce crystals in a high-temperature, high-pressure environment with an aqueous solution as the reaction medium. The hydrothermal method is suitable for the preparation of hydrophobic materials on a large scale, with inexpensive and simple reaction conditions.

It was reported a superhydrophobic wood surface with a contact angle of 152.9° was obtained by two-step hydrothermal synthesis of TiO_2_ solution and modification of fluoroalkylsilane on wood substrate [42]. However, the relatively effective superhydrophobic layer is too thin and poorly bonded at the interface between the wood and the coating, and therefore cannot withstand harsh conditions such as abrasion and corrosion, leading to hydrophobic instability and further degradation under the stimulus of the external environment [43]. Based on this, Tan et al. [44] prepared robust bulk superhydrophobic wood by a combination of hydrothermal and vacuum impregnation methods. Firstly, ZnO were synthesized in situ to build micro/nano-rough structures in the wood. Wood has low surface energy due to the substitution of hydroxyl groups on the surface of wood cells and zinc oxide by long-chain alkyl groups. The contact angle was 155° of the treated wood. The surface contact angle remains at approximately 150° for 180 s after sawing and abrasion. The superhydrophobic wood samples were immersed in hydrochloric acid (pH = 2, 1%), NaOH (pH = 12, 1%), n-hexane (95%), toluene (99.5%), ethanol (99.7%), acetone (99.5%), and DMF (99.5%) and treated for 48 h at room temperature. Petal-like fragments of ZnO were also distributed on the wood specimens after the chemical reagent treatment. The long-chain octadecyl isocyanate and polyfurfuryl alcohol, have good acid and base resistance properties. Therefore, the long-chain alkyl groups of palmitoyl chloride can cover zinc oxide and prevent it from being corroded by acid and alkali solutions, indicating that superhydrophobicity was present throughout the structure of the wood and provides excellent abrasion resistance, and chemical durability.

**Figure 6:**
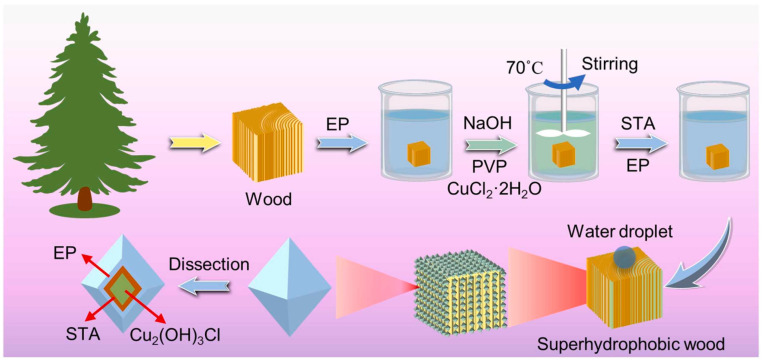
Scheme of the process used for preparation of superhydrophobic wood surface by EP/Cu_2_(OH)_3_Cl/STA/EP. Reproduced from [45], with permission from Elsevier, 2022.

Pinus wood was modified to make it superhydrophobic by a simple hydrothermal method for in situ synthesis of Cu_2_(OH)_3_Cl nanoparticles (NPs). Then, a stearic acid/epoxy resin (STA/EP) ethanol solution was impregnated in Figure 6 [45]. Modified wood exhibited superhydrophobicity, characterized by a WCA reach of 151 ± 3° and SA less than 6 ± 3°. The modified wood still remained superhydrophobic under harsh conditions including being impregnated in strong acid or alkali, 7 cycles of sandpaper abrasion, 200 cycles of tape paste, 192 h of UV irradiation, and exposed at 140 °C. The modified wood also exhibited excellent functions, including anti-mold, dimensional stability, stain resistance, and self-cleaning, which suggested that the process has potential for protecting wood in exterior exposures.

### 2.6. Etching

Etching methods include physical etching and chemical etching, which refers to a low surface energy treatment to achieve superhydrophobicity by roughening the surface of wood materials through laser or etching. It is usually combined with other methods to prepare superhydrophobic surfaces. The main etching methods include: chemical etching [46], plasma etching [47], laser etching [48,49], etc. The etching method allows precise manipulation and design on the substrate surface, but has a higher cost and is less economical.

Different rough surfaces of wood were constructed through fabrication of superhydrophobic wood surfaces by etching polydopamine coating with sodium hydroxide [50]. The results showed that the CA and SA of the NaOH-OTS were 151° and 4.8°, respectively. To test the mechanical stability of the samples, they were tested under continuous flow flushing. The superhydrophobic properties decreased slightly after 12 h, but were still close to the superhydrophobic level, the WCA was still over 150°. The roughness provided by the PDA overcame the weak interfacial binding problem between the inorganic nanoparticles and the organic matrix, thus showing a stronger resistance to loss. NaOH successfully etched the PDA coating, and the roughness was further improved by adding nano-SiO_2_. In addition, the superhydrophobic wood was obtained with high surface stability and anti-loss property, as well as resistance to acid and alkaline solutions and organic solvents.

### 2.7. Other Methods

In addition to the above methods, layer-by-layer assembly method was used to prepare a superhydrophobic wood surface coating. To prevent moisture deformation, water swelling, severe mildewing, and decay due to the biodegradation of wood outdoors. The method uses polydopamine (PDA) and 1H,1H,2H,2H-perfluoro-decyl trichlorosilane (PFDTS) [51]. PDA was used as an intermediate layer to connect the wood and PFDTS due to its self-adhesive properties. PDA was first deposited on the wood surface to form a highly crosslinked granular structure that improved the surface roughness of wood. The wood surface was further modified by low-surface-energy PFDTS, and the wood after PDA deposition was superhydrophobic with a WCA of 154°. The hydrophobic coating underwent rapid restoration and was resistant to both acidic and alkaline solutions with a pH of 2 to 12. 

The coating method is a simple and fast way to obtain differently shaped surfaces, including spraying method, dip-coating method, and brushing method. The spraying method is to use a spray gun to spray active particles into a mist and deposit them on the surface of the substrate to form a rough structure; dip-coating method is to soak the substrate in an active solution and deposit it to adhere to form a painted surface; brushing method is to apply the paint directly on the surface. It was researched that wood samples were dip-coated in a mixture of tung oil and natural beeswax, followed by the deposition of micronized sodium chloride particles by salt templating [52]. The result illustrated the successful formation of a micro-structured topography endowing the surface with water repellence as indicated by stable static water contact angles above 160°. It was thus a fully bio-based superhydrophobic wood coating achieved by dip coating.

Zhang et al. [53] explored an unconventional technique for the fabrication of superhydrophobic coatings. The stoichiometric reaction between OTS and water explored herein not only creates the required hierarchical micro/nanostructures but also results in low surface tension. This strategy is also conceptually different from conventional sol–gel processes. The superhydrophobic materials prepared in the study have excellent properties in terms of anti-icing, superhydrophobicity, scalability, and robustness for application on wood, fiber, paper, and plastic surfaces.

## 3. Application of Superhydrophobic Surfaces on Wood

With the development of bionics in various fields, wood science, and bionics have fused with each other. Wood bionics is the basis for the application of functional wood improvement technology, which is a cross-fertilization of wood science and bionics, aiming to build new functional bionic wood materials with high added value. The excellent properties of wood hydrophobic materials have led to the wide application of superhydrophobic wood due to its good characteristics, and have great potential applications in the fields of self-cleaning [54], double sparsity [55], self-healing, corrosion protection [56], oil–water separation [57,58], and intelligent constructions which have attracted a lot of attention from the academic and commercial fields.

### 3.1. Self-Cleaning Application

It lays down the bionic self-cleaning function of superhydrophobic materials for “Lotus unsullied from mud, wash clean without demon”. Superhydrophobic coating has a self-cleaning function for pollutants due to its wettability. Generally, to achieve self-cleaning with the help of rainwater washing and a certain tilt angle when there is dust, microorganisms, and other stains are deposited on the surface of a superhydrophobic coating. The sphere-like water droplets carry away contaminants by adsorption and thus achieve cleaning. Such self-cleaning superhydrophobic coatings can be applied to many surfaces in daily use, such as car windshields, windows, door glass, skyscrapers, solar panels, fabrics, sports shoes, metals, paper, sponges, wood, marble, etc. [59,60].

**Figure 7:**
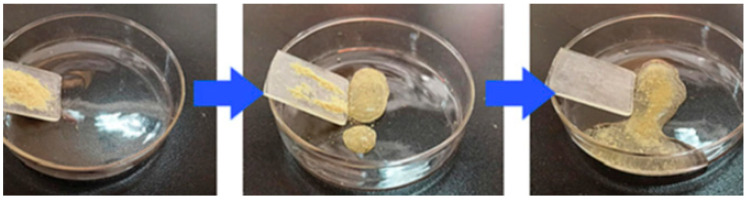
Self-cleaning test of the transparent wood. Reproduced from [66], with permission from Elsevier, 2023.

Superhydrophobic materials have the ability to self-clean with a sliding angle of less than 10° [61,62,63]. It was reported that superhydrophobic wood was fabricated by grafting poly(2-(perfluorooctyl)ethyl methacrylate) (PFOEMA) onto wood by atom transfer radical polymerization (ATRP) [64]. The resultant wood exhibited excellent water resistance with a WCA of 156° and SA of 4°. The modified wood also showed abrasion resistance, self-cleaning ability, and anti-mold properties, all of which are desirable for various wood products. In order to make full use of the self-cleaning properties of superhydrophobic wood for large-scale applications in ancient building preservation. The researchers used the method that involves synthesizing a two-component modifier solution consisting of SiO_2_ nanoparticles combined with poly(methylhydrogen)siloxane (PMHS) modification [65]. The superhydrophobicity of the coated surfaces exhibits excellent self-cleaning performance. The water droplets rolling on the superhydrophobic surface can remove the contaminants accumulated on the surface, mimicking the lotus leaf effect. For the modified wood surface, the water droplets readily rolled off, removing the methyl blue powder when the water dropped to the surface. The application of self-cleaning properties in transparent wood has attracted interest. The superhydrophobic and self-cleaning properties of transparent wood are of great significance for exploring lightweight building materials or electronic devices. In the study, the method of preparing superhydrophobic transparent wood (STW) was based on a delignified wood template [66]. The WCA of it was 163.5°, and the SA of the surface of STW was 5.2°, which could realize self-cleaning by hydration induction in Figure 7.

### 3.2. Self-Healing Applications

Self-healing superhydrophobic coatings have attracted much attention in recent years due to their unique self-retrieval properties and potential industrial applications [67,68]. The preparation of superhydrophobic wood with self-healing properties can effectively solve the durability problems during the use of wood, prolong the service life, and improve the abrasion resistance [69,70]. In general, there are two methods to achieve self-healing properties. The first one combines hydrophobic components with low surface energy and rough micro/nano structures. When the surface is damaged, the hydrophobic components migrate to the damaged area in response to external conditions, e.g., light [71], heat, or pH, thus restoring their hydrophobic properties [72,73,74]. 

It has been shown that an important indicator of self-healing ability for superhydrophobic wood material was examined by alkali etching [75]. After two hours of immersion in the alkali solution, the WCA of the surface decreased from 160° to 0°, indicating that the modified wood surface had lost its superhydrophobic properties. However, after exposing this damaged surface to the environment for 8 h, its WCA and SA recovered to 160° and <10°, respectively, indicating that the original superhydrophobicity of this damaged surface was restored. Wood pores that hold large amounts of fluoroalkyl silane as healing agents to recover their superhydrophobicity at room temperature when they migrated to the damaged surfaces. The etching–healing process could be repeated for nine cycles without reduction. Furthermore, it was found that the recovery time of superhydrophobicity was reduced to 2 h and 1 h when the temperature was increased to 100 °C and 140 °C, respectively. The self-healing process was faster at higher temperatures. For a shorter self-healing time of the superhydrophobicity coating, a newly found hydrophobic coating for wood achieves self-healing properties rapidly when damaged in acidic and alkaline environments [51]. When the wood was heated, the cross-linked fluorosilanes in the coating re-agglomerate to recover superhydrophobicity. The hydrophobic coating underwent rapid restoration and was resistant to both acidic and alkaline solutions (pH2–12). This study provides a simple way to endow superhydrophobic properties to wood and has potential multifunctional applications for wood protection and new products.

**Figure 8:**
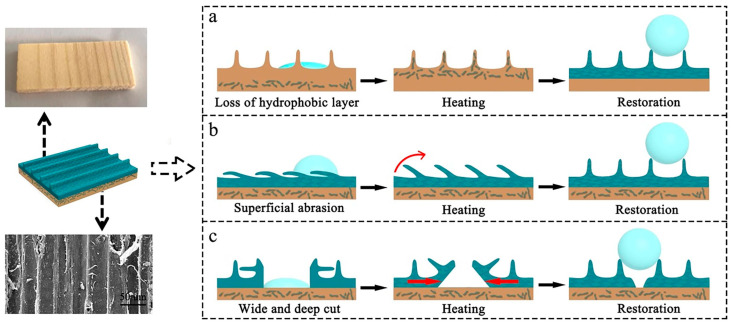
Schematic illustration of the healing behavior of the area after various damages. (**a**) Schematic illustration of the healing behavior. (**b**) Schematic illustration of the healing behavior of the area after superficial abrasion. (**c**) Schematic illustration of the healing behavior of the area after wide and deep cut. Reproduced from [78], with permission from Elsevier, 2019.

Second approach: recovery of hydrophobic properties is due to regeneration of the micro/nano hierarchy [76]. Self-recovering SHCs based on the recovery of structural damage have been successfully fabricated by spraying the mixture of polymethylmethacrylate(PMMA)/zinc stearate(ZNO)/stearic acid(STA) on the wood surface [77]. The damaged coatings were soaked in water, followed by the infiltration of water into the damaged structure, and then induced swell of PMMA to restore the topographic features which were compressed during scouring. When the water in the coatings is evaporated and leaves the air hole again; therefore, the coatings recover the superhydrophobicity. By acid rain (pH ≈ 3.5) scouring, the coatings restored superhydrophobicity after immersion in water for 30 min. Coatings are still superhydrophobic within the process repeated four times. In contrast to most conventional self-healing surfaces, the superhydrophobic surface prepared by Jia et al. could restore wide and deep cuts by the healable conveyance method [78]. By assembling layered bismuth oxychloride (BiOCl) nanomaterials and hydrophobic perfluorooctyltriethoxysilane (PFOTS) layers on a wood substrate, PFOTS molecules could break the energy gap and migrate to the damaged surface by a simple heating treatment. Then, PFOTS molecules readily reacted with hydroxyl groups of ethanol and regenerated a new hydrophobic PFOTS layer. The fibers could deliver the superhydrophobic coating to the damaged area in Figure 8. In this way, the incision was reduced and filled, thus regenerating the superhydrophobicity. The obtained self-healing superhydrophobic surfaces with photocatalytic properties should stimulate the development of multifunctional intelligent materials for applications in related fields.

### 3.3. Oil–Water Separation Application

Oil spills are considered to be one of the major pollutions in the field of water pollution today. It becomes a challenging issue for oil spill management. Wastewater contains a variety of oils from industrial accidental oil spills, including those that occur when oil is transported by truck or ship. These can cause damage to land, rivers, and oceans. Potential applications for superhydrophobic surfaces are to separate oil from water to reduce pollution [79]. Wood is an excellent matrix for creating these surfaces because its interactive chemistry and intricate cellular matrix provide a large, reactive surface area for fabrication.

**Figure 9:**
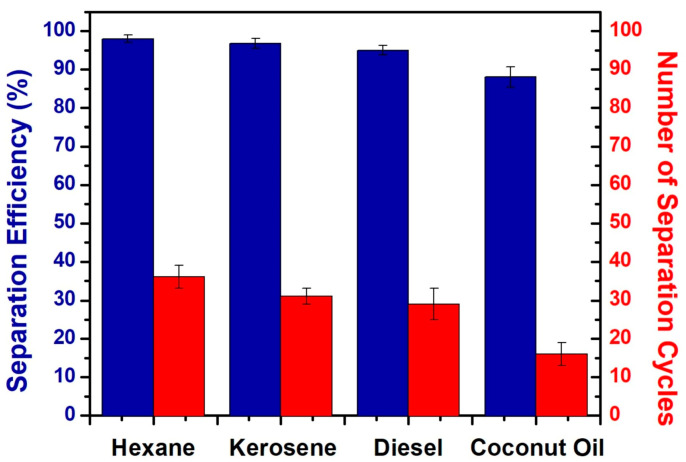
Separation efficiency and recycle ability of various oil–water mixtures by the superhydrophobic SP-3 pellet. Reproduced from [80], with permission from Elsevier, 2020.

**Figure 10:**
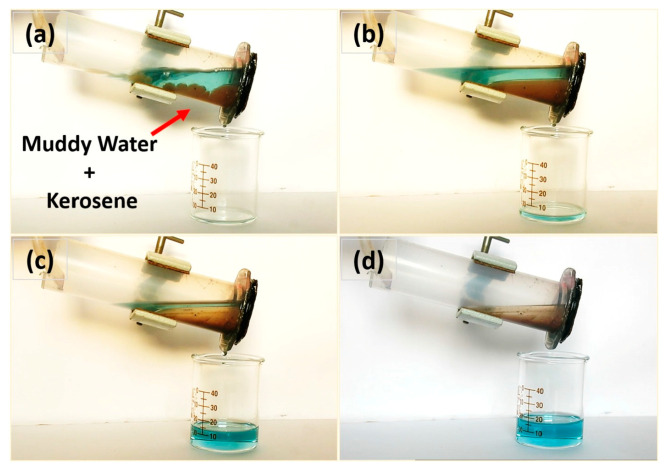
Muddy water—kerosene separation by superhydrophobic SP-3 pellet (**a**) before separation; (**b**) after 3 min of separation; (**c**) after 8 min of separation; (**d**) after 12 min of separation. Reproduced from [80], with permission from Elsevier, 2020.

In addition to traditional superhydrophobic materials, new materials are used for efficient oil–water separation, such as candle soot, corn stover fiber, and wood chips. Biodegradable wood chips are one of the emerging materials for superhydrophobic oil–water separation applications at present. It was reported that [80] utilized a low-cost sawdust–polystyrene (SD–PS) composite and developed a facile strategy to prepare a free-standing superhydrophobic pellet for efficient oil–water separation. More importantly, it is feasible to simple recovery of the absorbed oil. The results expressed that the fabricated superhydrophobic pellet can show excellent separation efficiency for various oils from water, including turbid water, warm water, also for kerosene, n-hexane, diesel, and coconut oil (Figure 9 and Figure 10).

Wood-based superhydrophobic surfaces could represent a more environmentally benign material for remediating spills. Na_3_(Cu_2_(CO_3_)_3_OH)∙4H_2_O was synthesized in situ on delignified balsa wood reacting copper chloride and sodium hydroxide in the presence of phenol formaldehyde (PF) resin, and then modifying the surface to be superhydrophobic with stearic acid (STA) [81]. The modified wood had excellent absorption and filtration capabilities for various oils and was able to absorb 2.1–4.8 times its weight in oil, with a maximum oil absorption of 5.2 g/g for chloroform. The modified wood could be regenerated and reused up to 14 times, and the still retained a separation efficiency of 90% for the dichloromethane: water mixture within 11 cycles. Herein [82], a superhydrophobic elastomer was reported. The most important finding is that the unique micro/nanostructure in the original wood was well maintained in the final wood elastomer for solar-assisted crude oil recovery as well as water/oily liquids separation.

### 3.4. Other Applications

**Figure 11:**
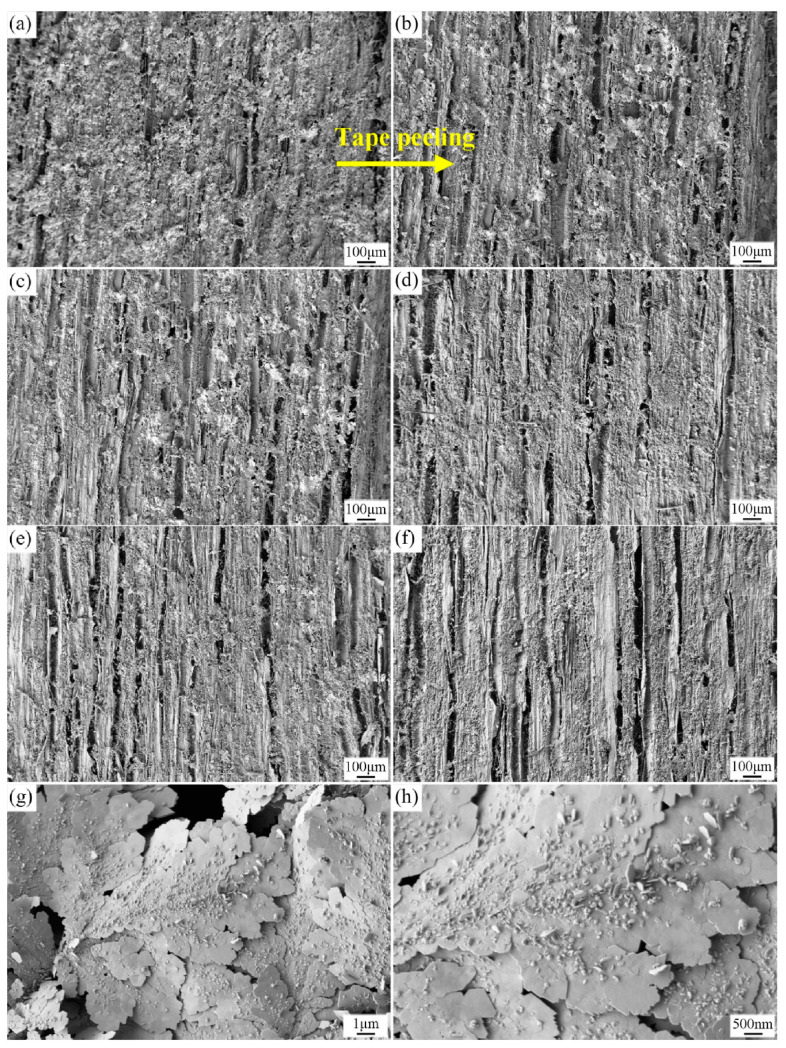
SEM images of the superhydrophobic wood surface after tape peeling for (**a**) 0, (**b**) 20, (**c**) 40, (**d**) 60, (**e**) 80, and (**f**) 100 cycles. The magnified images of (**f**) are shown in (**g**,**h**). Reproduced from [83], with permission from Elsevier, 2021.

The mechanical durability of superhydrophobic wood surfaces is of practical importance for civil engineering and construction. It has UV resistance, superhydrophobicity, self-cleaning, and stain resistance. It has great potential to replace non-biodegradable fossil-based materials in the construction field due to its ease of preparation, biodegradability, durability, and environmental friendliness which are of great interest. Superhydrophobic wood surfaces with robust mechanical durability were fabricated by a non-toxic chemical solution of silver nitrate (AgNO_3_) [83]. The WCA slowly decreased from 160.5° to 156.5° during the cyclic tape peeling test. The variation range was quite small. Figure 11 displays SEM images of the surface under tape peeling for different cycles. When the number of peelings reaches up to 100, the structures look less rough, but from the enlarged image, it can be seen that the hierarchical micro/nano dendritic structures have not changed. Abrasion tests with sandpaper were performed on the coated surface with a weight of 50 g, and the WCA was reduced by only about 3° when the abrasion length reached 200 cm, proving the robustness of the superhydrophobic surface layer. Water droplets also rolled off the scratched area easily when the surface was scraped arbitrarily with a knife. This mechanically durable superhydrophobic wood has promising applications in civil engineering and construction.

The prepared poplar reconstituted wood exhibited excellent mechanical durability, good chemical stability, and excellent self-cleaning and anti-fouling abilities. Jiang et al. [84] synthesized pine-cone-shaped Cu_7_Cl_4_(OH)10·H_2_O nanoparticles in situ on the radial cross-section of poplar reconstituted wood surface by hydrothermal method. It provides a new pathway for the superhydrophobic modification of wood-based engineering materials, which has the potential to be applied to the industrial production of superhydrophobic wood structures and promote the sustainable development of wood resources. Versatile and stable superhydrophobic and highly optically transparent superhydrophobic surfaces are of increasing interest to researchers. Here, Yang et al. [85] synthesized bionic robust superhydrophobic transparent coatings with nanostructures based on polyethylene glycol functionalized SiO_2_/PVA/PAA/fluoropolymer coatings, and the bionic robust superhydrophobic wood is superhydrophobic has high- and low-temperature resistance, and has high water repellency.

## 4. Conclusions

Under the global trend of carbon peaking and carbon neutrality, as a typical natural material with carbon sequestration properties during its life cycle, wood has been widely used in construction, furniture, and energy production as well as other functional materials due to its layered and porous structure. In order to further improve the physical and mechanical properties of natural wood, hydrophobic modifications are applied to wood to extend its service life and broaden its application areas. Superhydrophobic coatings have great potential for application in the field of wood. Although research on superhydrophobic wood has been going on for many years, there are still problems with the durability of superhydrophobic coating surfaces, mechanical resistance, etc.

(1) It is necessary to develop simple and feasible preparation processes that are economical and environmentally friendly.

In the preparation of superhydrophobic coatings on wood, the graft copolymerization method takes longer preparation time. The demanding harsh reaction conditions of the CVD method have limited the large-scale application in the preparation of superhydrophobic wood. The etching method has a higher cost and is less economical. In addition, there are modification reagents that are not friendly to the environment. Therefore, a preparation method of superhydrophobic wood with a simple preparation process, low cost, and suitable for large-area preparation is also needed.

(2) Improving the mechanical resistance.

The mechanical strength of superhydrophobic wood includes two aspects. One is the interfacial bond between the superhydrophobic coating and the substrate. Another one is the mechanical strength of the micro/nanostructure on the surface of the superhydrophobic wood. During processing and use, poor bonding between superhydrophobic coatings and substrates can lead to their peeling off, and low surface energy substances on the surface of superhydrophobic wood can easily decompose under the stimulation of temperature, light, and strong oxidants. The micro/nanostructure is damaged by physical action. This all affects the hydrophobic properties of superhydrophobic woods. It is a challenge to find superhydrophobic coatings that are easy to manufacture and robust in practical applications.

(3) A comprehensive standard system is imperative for the evaluation and comparison the of the durability of wood superhydrophobic coatings.

Under the rapid development in the field of superhydrophobic wood materials, the definition of coating parameters and testing procedures has not yet been standardized. So far, there is no unified test method including test conditions and evaluation criteria to compare artificial superhydrophobic coatings.

## 5. Future Prospects

With the development of new materials and the emergence of new technologies, it is always essential to manufacture new durable and functional superhydrophobic wood materials. The following perspectives are proposed for future research on superhydrophobic materials.

(1) Improving and optimizing existing methods for preparing superhydrophobic wood coatings. Formation of chemical bonds between wood and hydrophobic surfaces to enhance the bonding force, and at the same time, simultaneous application of multiple preparation methods according to its advantages. Devoted to exploring simple, energy-saving, and environmentally friendly methods for preparing wood superhydrophobic to further develop strong and low-cost superhydrophobic wood.

(2) More application areas should be further explored to give wood materials functionalization, develop multi-functional and intelligent wood, and apply them to intelligent home architecture and other fields, such as transparent wood, magnetic wood, and new intelligent materials for energy storage and conversion. It is of great significance for the future of green and sustainable development.

## Data Availability

Not applicable.
